# Detection of ribophagy in yeast and mammals

**DOI:** 10.52601/bpr.2024.240002

**Published:** 2024-04-30

**Authors:** Miao Ye, Yuting Chen, Zhaojie Liu, Yigang Wang, Cong Yi

**Affiliations:** 1 Xinyuan Institute of Medicine and Biotechnology, School of Life Sciences and Medicine, Zhejiang Sci-Tech University, Hangzhou 310018, China; 2 Department of Biochemistry, and Department of Hepatobiliary and Pancreatic Surgery of the First Affiliated Hospital, Zhejiang University School of Medicine, Hangzhou 310058, China; 3 Guangzhou Municipal and Guangdong Provincial Key Laboratory of Protein Modification and Degradation, Affiliated Cancer Hospital & Institute of Guangzhou Medical University, School of Basic Medical Sciences, Guangzhou Medical University, Guangzhou 510182, China

**Keywords:** Ribophagy, Ribosome turnover, Immunoblotting, Fluorescence microscopy, Transmission electron microscopy, Yeast and mammals

## Abstract

Ribophagy, the cellular process wherein ribosomes are selectively self-digested through autophagy, plays a pivotal role in maintaining ribosome turnover. Understanding the molecular regulatory mechanisms governing ribophagy is pivotal to uncover its significance. Consequently, the establishment of methods for detecting ribophagy becomes important. In this protocol, we have optimized, enriched, and advanced existing ribophagy detection techniques, including immunoblotting, fluorescence microscopy, and transmission electron microscopy (TEM), to precisely monitor and quantify ribophagic events. Particularly noteworthy is the introduction of TEM technology for yeast ribophagy detection. In summary, the delineated methods are applicable for detecting ribophagy in both yeast and mammals, laying a solid foundation for further exploring the physiological importance of ribophagy and its potential implications in diverse cellular environments.

## INTRODUCTION

Ribosome biogenesis and protein synthesis are fundamental, rate-limiting steps crucial for cell growth and proliferation (Kang *et al*. 2021). In a typical human cell, ribosomal proteins constitute approximately 4%–6% of the total protein mass (Shore *et al.* 2022). Hence, ribosome turnover is critical for maintaining cellular homeostasis (Ni and Buszczak 2023). Ribophagy, a selective autophagy process, involves targeted degradation of ribosomes, and assumes a pivotal role in this turnover process, ensuring the removal of damaged or surplus ribosomes to maintain cellular efficiency (Cebollero *et al.* 2012). This selective degradation serves as a dynamic response to various cellular conditions, including stress and nutrient availability (Lafontaine 2010). The regulation of ribophagy entails intricate molecular mechanisms, involving autophagy-related pathways and specific recognition factors for damaged ribosomes. In mammals, NUFIP1 has been identified as a ribophagy receptor responsible for the autophagic degradation of ribosomes in response to starvation (Wyant *et al.* 2018). In *Saccharomyces cerevisiae*, although no ribophagy receptor has been found yet, the Ubp3p/Bre5p ubiquitin protease has been identified as required for yeast ribophagy (Kraft *et al.* 2008). Based on recent advances in the field of ribophagy and a better study of the molecular mechanisms governing this process, we have refined, expanded, and elevated existing ribophagy detection techniques to assess the extent of ribophagy occurrence in response to various stimuli.

### Development of the protocol

The protocol is primarily structured into four parts: imaging detection of yeast and mammalian ribophagy (Yi *et al*. 2012; Yu *et al*. 2010); Western blot detection of ribophagy in yeast and mammals (Wyant *et al.* 2018; Kraft *et al*. 2008); detection of Yeast and mammalian ribosomal RNA (rRNA) (Shedlovskiy *et al*. 2017; Li *et al*. 2021); and TEM detection for yeast and mammalian ribophagy (Wyant *et al.* 2018; Li *et al*. 2022). The protocol provides a comprehensive and systematic procedure for detecting the levels of ribophagy occurrence in both yeast and mammalian cells. To enhance the observation of ribophagy in fluorescence imaging, it's essential to knockout the *ATG15* gene in yeast cells, thus preventing the degradation of autophagic bodies within vacuoles (Yorimitsu *et al.* 2005). In mammalian cells, the addition of Concanamycin A serves to inhibit the degradation of autophagic substrates in autolysosomes (Wyant *et al.* 2018). Furthermore, in the assessment of ribophagy occurrence in yeast through TEM observation, we have innovatively employed APEX2 tag to label the ribosomal large subunit Rpl10 in the *pep4*∆ yeast cells (Pep4 is the vacuolar acidic hydrolase in yeast, and its knockout inhibits the degradation of proteins within the vacuole), successfully establishing a TEM detection protocol for yeast ribophagy (Li *et al*. 2022). In summary, this detailed and improved protocol is useful to study the molecular regulatory mechanisms and physiological functions associated with ribophagy in yeast and mammalian cells.

### Applications and advantages of the protocol

This protocol offers a systematic approach from the perspectives of biochemistry, cell biology, and structural biology to evaluate the occurrence level of ribophagy in both yeast and mammalian cells. Not confined to ribophagy alone, it can also be extended to investigate other selective autophagy processes in yeast and mammalian cells by labeling various organelle marker proteins.

### Limitations of the protocol

(1) The protocol for detecting the occurrence level of ribophagy described here is only suitable for yeast and mammalian cells. For protein extraction, rRNA extraction, and TEM pre-treatment in other species, alternative methods suitable for those organisms should be followed.

(2) The extent of ribophagy occurrence induced by various stimuli and treatment durations can differ significantly. Adjustments in specific induction conditions and treatment durations should be made according to the purpose of the experiment.

(3) During the process of rRNA extraction, it is necessary to minimize RNA degradation as much as possible.

## OVERVIEW OF THE PROTOCOL

### Assessing ribophagy in both yeast and mammalian cells using a fluorescence microscope

#### Assessing yeast ribophagy through a fluorescence microscope

(1) The relevant yeast strains are taken out from the −80 °C freezer and streaked onto corresponding solid agar plates for activation.

(2) The relevant yeast cells are grown in the corresponding liquid culture medium until reaching an OD_600_ = ~1.0. Fluorescence microscopy is used to observe the extent of ribophagy, wherein ribosomal proteins tagged with GFP enter the vacuole (Janke *et al.* 2004).

(3) Subject yeast cells in the logarithmic growth phase to nitrogen starvation. After 6 h of starvation, fluorescence microscopy was used to observe the extent of ribophagy, wherein ribosomal proteins tagged with GFP enter the vacuole under nitrogen starvation conditions.

#### Assessing mammalian ribophagy through laser confocal microscopy

(1) U2OS WT and ATG7 KO cells were taken out from the liquid nitrogen tank for recovery (Qian *et al*. 2023).

(2) When the cell density reaches 90%, U2OS WT and ATG7 KO cells are subjected to EBSS starvation for either 0 h or 10 h. Subsequently, specific ribosomal protein and lysosomal marker protein Lamp2 double immunofluorescent staining is conducted on cells.

(3) Observing the level of ribophagy occurrence in U2OS WT and ATG7 KO cells using laser confocal microscopy.

### Examining ribophagy in yeast and mammalian cells by Western blot analysis

#### Examining yeast ribophagy through Western blot analysis

(1) The relevant yeast strains are taken out from the −80 °C freezer and streaked onto corresponding solid agar plates for activation.

(2) The relevant yeast cells are grown in the corresponding liquid culture medium until reaching an OD_600_ = ~1.0. Collect five OD_600_ units of yeast cells, centrifuge, discard the supernatant, and store the pellet at −80 °C.

(3) Subject yeast cells in the logarithmic growth phase to nitrogen starvation. After six hours of starvation. Collect five OD_600_ units of yeast cells, centrifuge, remove the supernatant, and store the pellet at −80 °C.

(4) Extract yeast proteins using the NaOH method for Western blot analysis (Kushnirov 2000). Evaluate the occurrence level of ribophagy by detecting the GFP cleavage of ribosomal proteins tagged with GFP.

#### Examining mammalian ribophagy throughWestern blot analysis

(1) U2OS WT and ATG7 KO cells were taken out from the liquid nitrogen tank for recovery.

(2) When the cell density reaches 90% in a 6-cm dish, U2OS WT and ATG7 KO cells are subjected to EBSS starvation treatment for 10 h.

(3) Extract mammalian cell proteins by adding protein loading buffer to the cell pellet for Western blot analysis. Evaluate the occurrence level of mammalian ribophagy by detecting the degradation of endogenous ribosomal proteins.

### Evaluating the levels of rRNA in both yeast and mammalian cells through RNA extraction

#### Evaluating yeast rRNA through RNA extraction

(1) The relevant yeast strains are taken out from the −80 °C freezer and streaked onto corresponding solid agar plates for activation.

(2) The relevant yeast cells are grown in the corresponding liquid culture medium until reaching an OD_600_ = ~1.0. Collect five OD_600_ units of yeast cells, centrifuge, discard the supernatant, and store the pellet at −80 °C.

(3) Subject yeast cells in the logarithmic growth phase to nitrogen starvation. After six hours of starvation. Collect five OD_600_ units of yeast cells, centrifuge, discard the supernatant, and store the pellet at −80 °C.

(4) Extract yeast total RNA. Evaluate the level of yeast rRNA by running RNA agarose gel.

#### Evaluating mammalian rRNA through RNA extraction

(1) U2OS WT and ATG7 KO cells were taken out from the liquid nitrogen tank for recovery.

(2) When the cell density reaches 90% in a 6-cm dish, U2OS WT and ATG7 KO cells are subjected to EBSS starvation for 10 h.

(3) Extract total RNA from an equal number of mammalian cells. Evaluate the level of mammalian rRNA by running RNA agarose gel.

### Detecting ribophagy in yeast and mammalian cells using TEM

#### Detecting yeast ribophagy using TEM

(1) The relevant yeast strains are taken out from the −80 °C freezer and streaked onto corresponding solid agar plates for activation.

(2) The relevant yeast cells are grown in the corresponding liquid culture medium until reaching an OD_600_ = ~1.0. Collect 20 OD_600_ units of yeast cells, centrifuge, remove the supernatant, and the pellet was fixed with 2% paraformaldehyde and 2.5% glutaraldehyde in PBS. After resuspending the yeast cells by pipetting, shake them at 200 r/min at 30 °C for 30 min, followed by overnight incubation at 4 °C.

(3) Subject yeast cells in the logarithmic growth phase to nitrogen starvation. After six hours of starvation. Collect 20 OD_600_ units of yeast cells, centrifuge, remove the supernatant, and the yeast cells are fixed using the same method as described above.

(4) After fixation of the yeast cells, sample preparation is carried out following the detailed steps for yeast TEM analysis. Following sample preparation, use TEM to detect the level of yeast ribophagy occurrence.

#### Detecting mammalian ribophagy using TEM

(1) U2OS WT and ATG7 KO cells were taken out from the liquid nitrogen tank for recovery.

(2) When the U2OS WT and ATG7 KO cell density reaches 90% in a 6-cm dish, cells are subjected to EBSS starvation for 10 h.

(3) Cells were fixed overnight with 1 mL 2.5% glutaraldehyde in PBS.

(4) After fixation of the mammalian cells, sample preparation is carried out following the detailed steps for mammalian TEM analysis. Following sample preparation, use TEM to detect the level of mammalian ribophagy occurrence.

## PROCEDURE

### Validation of yeast ribophagy using a fluorescence microscope

#### Cell culture [TIMING 4 d]

1 Activation. Yeast strains: BY4741-Vph1-mCherry::*KanMX6-atg15*Δ::*hphNT1-*Rpl10-GFP::*HisMX6*, BY4741-Vph1-mCherry::*KanMX6-atg5*Δ::*hphNT1-*Rpl10-GFP::*HisMX6*, BY4741-Vph1-mCherry::*KanMX6-atg15*Δ::*hphNT1-*Rps27a-GFP::*HisMX6*, and BY4741-Vph1-mCherry::*KanMX6 atg5*Δ::*hphNT1-*Rps27a-GFP::*HisMX6* are taken out from the –80°C freezer and streaked onto SD-His agar plates. Subsequently, these yeast strains were inoculated into glass tubes containing SD-His medium supplemented with the specific antibiotics (Hygromycin B, 200 mg/mL, 1:1000; G418, 250 mg/mL, 1:1000), and the prepared glass tubes were placed in a shaking incubator set at 30 °C and 230 r/min for 12 h.

2 Cultivation. After reaching saturation in growth, an adequate volume of yeast was transferred to a conical flask containing 15 mL of SD-His medium, starting at an OD_600_ = 0.25. This flask was then placed in a shaking incubator at 30 °C, 230 r/min for about 5 h, allowing yeast cells to reach the logarithmic growth stage (OD_600_ = 1.0–1.2).

#### Yeast starvation [TIMING 7 h]

3 Nitrogen starvation. After the yeast cells were grown to the logarithmic growth stage (OD_600_ = 1.0–1.2), a 200 μL yeast sample was taken for observation under fluorescence microscopy. The remaining yeast culture was centrifuged at 4000 r/min for 2 min, discarding the supernatant. The remaining precipitate was washed twice with 5 mL of SD-N medium. Following these washes, the yeast cells were resuspended in 15 mL of SD-N medium (OD_600_ = 1.0–1.2). The flasks were then placed on a shaking incubator at 30 °C, 230 r/min. 200 μL yeast sample was taken for fluorescence microscopy analysis following nitrogen starvation treatment for 6 h.

#### Microscopic observation [TIMING 1 h]

4 Observation. Yeast cells were examined utilizing a 100× oil microscope, with GFP excited by a 488-nm laser and mCherry excited by a 560-nm laser.

### Validation of mammalian ribophagy with a laser confocal microscope

#### Cell culture [TIMING 3–5 d]

5　Cell resuscitation.

(A) Thawing. U2OS WT and ATG7 KO cells were retrieved from the liquid nitrogen and quickly thawed in a 37 °C water bath.

(B) Collection. The cell suspension was then transferred to a 1.5 mL centrifuge tube and spun at 1000 r/min for 3 min.

(C) Washing. After discarding the supernatant, the cells were resuspended in 1 mL of DMEM medium and centrifuged again at 1000 r/min for 3 min.

(D) Cultivation. Following supernatant removal, 1 mL of DMEM medium was added for cell resuspension. The cell suspension was transferred to a 6-cm dish and supplemented with 2 mL of DMEM medium. The mixture was gently agitated, and the cells were cultured at 37 °C in a 5% CO_2_ cell incubator.

**[CRITICAL STEP]** DMEM medium contains 10% FBS and 1% penicillin-streptomycin.

6　Cell passage.

(A) Washing. Upon reaching approximately 90% cell density, the DMEM medium was removed, and the cells were rinsed once with 1 mL of PBS.

(B) Digestion. The cells underwent digestion with 0.25% Trypsin-EDTA at 37 °C for 3 min. Subsequently, the 0.25% Trypsin-EDTA was discarded, and the cells were rinsed with 1 mL of DMEM medium. The cell suspension was collected in a 1.5-mL centrifuge tube by centrifugation at 1000 r/min for 3 min.

(C) Cultivation. Following the removal of the supernatant, the cells were washed with 1 mL of PBS at 1000 r/min for 3 min. Next, 1 mL of DMEM medium was used to re-suspend the cells. The transferred cell suspension was then cultured in a 10-cm dish containing 7 mL of DMEM medium at 37 °C in a 5% CO_2_ cell incubator.

#### Immunofluorescence [TIMING 4 d]

7　Cell preparation.

(A) Washing. Upon reaching approximately 90% cell density, the digestion and collection of cells are performed like cell passage. In short, discard the cell medium and wash the cells with 3 mL of PBS.

(B) Digestion. Treat cells with 1 mL 0.25% trypsin-EDTA, and halt the digestion process by adding 3 mL of DMEM medium. Subsequently, collect the cells in a 15-mL centrifuge tube by centrifuging at 1000 r/min for 3 min, and resuspend the cells with 1 mL of DMEM medium.

(C) Cultivation: Transfer cell aliquots into 12-well dishes, each containing a coverslip, and add 1 mL of DMEM medium to each well. Approximately 10,000 cells were then added to each well, thoroughly mixed, and cultured at 37 °C in a 5% CO_2_ cell incubator for 24 h.

8 Cell starvation. The medium was discarded, and the cells were washed twice with 1 mL of PBS, and then treated with 1 mL of EBSS medium for 10 h. Additionally, supplement the EBSS medium with 500 nmol/L Concanamycin A.

9 Cell fixation. The medium was discarded, and the cells were washed three times with 1 mL of PBS. Subsequently, the cells were fixed using 1 mL of 4% PFA for 15 min at room temperature (RT).

10 Blocking. The cells were washed three times in 1 mL PBS, followed by a blocking solution prepared in 1 mL PBS with 0.1% Triton X-100 and 5% FBS for 2 h at RT.

11 Primary antibody incubation. Following three washes with 1 mL PBS, 50 μL of primary antibody (anti-RPL7 (1:300, 14583-1-AP, Proteintech) and anti-LAMP2 (1:1000, 27823-1-AP, Proteintech); anti-RPS5 (1:300, 16964-1-AP, Proteintech) and anti-LAMP2 (1:1000, 27823-1-AP, Proteintech)) prepared in the blocking solution was placed onto a transparent film. Using tweezers, cells were delicately lifted, excess PBS was removed, and the cells were carefully positioned on the primary antibody, then incubated at RT for 2 h.

12 Secondary antibody incubation. The cell-attached coverslips were gently returned to the 12-well plate. After three washes with 1 mL PBS, 50 μL of the secondary antibody (Goat anti-Mouse IgG H&L Alexa Fluor® 488 (1:1000), ab150113, Abcam; Goat anti-Rabbit IgG H&L Alexa Fluor® 594(1:1000), ab150080, Abcam) prepared in the blocking solution, was applied onto the transparent film. The cell-attached coverslips were cautiously lifted using tweezers, excess PBS was removed, and the coverslips were inverted onto the secondary antibody, then incubated at RT for 1 h.

13 Cell sealing plate.

(A) Washing. Utilizing tweezers, the cell slides were carefully placed back into the 12-well plate and washed three times with 1 mL of PBS.

(B) Sealing. 10 μL of the anti-fade mounting medium was added to the slide. Gently remove the cell attachment with tweezers, wipe off PBS, place the cell face down in the anti-fade mounting medium, and apply nail polish along the edge of the cell attachment to stabilize it.

(C) Storage. Store overnight at RT, shielded from light. The following day, either use Nikon confocal microscopy or store in a film box at 4 °C.

14 Imaging. A Nikon laser confocal microscope was used for observation. Adjust the intensity of 488 laser wavelength to 6 and the Gain to 45. For 561 laser wavelength, adjust the intensity to 3, and the Gain to 35.

### Validation of yeast ribophagy with Western blot analysis

#### Cell culture [TIMING 4 d]

15 Activation. Yeast strains: BY4741-Rpl10-GFP::*HisMX6*, BY4741-*atg1*Δ::*hphNT1-*Rpl10-GFP::*HisMX6*, BY4741-Rps27a-GFP::*HisMX6*, and BY4741-*atg1*Δ::*hphNT1-*Rps27a-GFP::HisMX6. The experimental procedure is the same as Step 1.

16 Cultivation. The experimental procedure is the same as Step 2.

#### Yeast treatment and collection [TIMING 7 h]

17 Collection. After the yeast reached the logarithmic growth stage, five OD_600_ yeast cells were transferred to a 15 mL centrifuge tube and spun at RT at 4000 r/min for 2 min. Following the removal of the supernatant, the precipitate was washed with 700 μL of pre-cooled deionized water, and the yeast was then transferred to a 1.5 mL microtube, centrifuged again at RT, 12,000 r/min for 1 min, and the supernatant was discarded, and the pellet was stored at −80 °C.

**[CRITICAL STEP]** Sample collection should be operated on ice.

18 Nitrogen starvation. The remaining yeast cells were centrifuged at 4000 r/min for 2 min. After discarding the supernatant, the pellet underwent two washes with 5 mL of SD-N medium, and 10 mL of SD-N medium was added for resuspension. The tubes were then placed in a shaking incubator at 30 °C, 230 r/min for 6 h.

19 Collection. Following 6 h of nitrogen starvation, five OD_600_ yeast cells were collected using the same method described in Step 17.

#### Sample preparation for Western blot [TIMING 30 min]

20 Add 200 μL of 0.1 mol/L NaOH to the collected yeast cells and mix thoroughly. Subsequently, place the centrifuge tube at 30 °C for 5 min, then centrifuge at 12,000 r/min for 1 min. Discard the supernatant, add 100 μL of 2× SDS sample buffer, and mix well. Finally, place the centrifuge tube at 95 °C for 5 min.

**[CRITICAL STEP]** Avoid using a vortexer for mixing, use a pipette to mix the samples. These prepared samples can be stored at −20 °C for Western blot.

#### SDS-PAGE Gel [TIMING 2 h]

21 Prepare the protein electrophoresis equipment along with a 10% polyacrylamide gel. Load 15 μL of protein samples and a protein marker into the gel wells. Initiate electrophoresis at 90 V until the proteins enter the separating gel. Then, adjust the voltage to 140 V, and allow the electrophoresis to run for approximately 1.5 h.

#### Western blot [TIMING 1 d]

22 Transfer. Set up the protein transfer equipment and ensure the nitrocellulose membranes are fully immersed in the transfer buffer. Assemble the polyacrylamide gel, nitrocellulose membranes, and transfer clips. Transfer the membranes for 1.5 h at 250 mA.

23 Block. Submerge the transferred membrane in a container with 10 mL of PBST for rinsing. Then, place it in another container with 25 mL of 5% skimmed milk. Place the container on a shaker at 40 r/min for 20 min. Afterward, transfer the membrane to another container with 10 mL of PBST, using a shaker at 100 r/min for 2 min. Repeat this washing step once more.

24 Primary antibody incubation. The washed membrane was immersed in primary antibody (anti-GFP antibody, 1:3000; anti-Pgk1 antibody, homemade, 1:5000; diluted with primary antibody dilution buffer) for incubation.

25 Washing. Upon completion of incubation, wash the membrane three times with 10 mL of PBST for 10 min each time.

26 Secondary antibody incubation. Following the washing steps, immerse the membrane in the secondary antibody solution (anti-Rabbit antibody, 1:10000, diluted with 5% skimmed milk in PBST) for incubation.

**[CRITICAL STEP]** Antibody incubation can be incubated at 4 °C overnight or at RT for 1 h.

27 ECL. Mix the enhanced chemiluminescence solution A and B at a 1:1 ratio. Apply this mixture evenly onto the membrane and then place it in a developer to initiate the development process.

28 Western blot data analysis.

### Validation of mammalian ribophagy by Western blot analysis

#### Cell culture [TIMING 3–5 d]

Cell resuscitation and passage

29 Cell preparation was conducted as described in Steps 5–7. The cells were then added to each well of a 12-well plate and cultured for 24 h at 37 °C with 5% CO_2_.

#### Cell starvation [TIMING 10 h]

30 Discard the medium, wash the cells twice in 1 mL PBS, and add 1 mL EBSS medium within 10 h.

#### Sample collection and Western blot [TIMING 1 d]

31 The medium was discarded, and the cells were washed once in 1 mL PBS. 300 μL of 2× SDS loading was added to collect the cells. The cell suspension was transferred to a 1.5 mL centrifuge tube and heated at 95 °C for 10 min. Finally, 10 μL of each sample was used for Western blot analysis to assess the degradation level of endogenous ribosomal proteins using the indicated antibodies (anti-Rpl26, anti-Rpl7, anti-Rps5, and anti-Rps14).

### Extraction of total RNA in yeast cells

#### Cell culture [TIMING 1 d]

32 Inoculation. Yeast strains: BY4741-*pep4*Δ::*KanMX6*, BY4741-*pep4*Δ::*KanMX6-atg1*Δ::*hphNT1*. The experimental procedure is the same as Step 1.

33 Cultivation. The experimental procedure is the same as Step 2.

#### Yeast treatment and collection [TIMING 7 h]

34 Sample collection. The experimental procedure is the same as Step 17.

**[CRITICAL STEP]** Sample collection should be operated on ice.

35 Nitrogen starvation. Sample treatment procedure was described in Step 18.

36 The sample collection. The experimental procedure is the same as Step 19.

#### FAE Extraction of yeast total RNA [TIMING 1h]

37 Resuspend the yeast pellets in FAE using a pipette or vortex, ensuring a minimum of 30–40 μL of FAE per 1 OD_600_ unit of yeast.

38 Heat the cell suspension in FAE at 70 °C for 10 min.

39 Vortex the mixture briefly, then centrifuge it at 12,000 r/min in a tabletop microcentrifuge at RT for 2 min.

40 Carefully transfer the FAE supernatant containing RNA to a fresh RNase-free microtube, leaving about 5–10 μL of supernatant remaining on top of the cell pellet to prevent cell carryover.

41 Storage. Take a small amount for testing (3–5 μL), and store the remainder at −80 °C for preservation.

42 RNA gel. Mix the supernatant with 6× RNA loading buffer, and subsequently, load 10 μL of the mixture onto the RNA gel. Run the gel at 150 V for 15 min. After the run, analyze the content and composition of the RNA samples using a gel imaging system to observe the results.

### Extraction of total RNA in mammalian cells

#### Cell culture [TIMING 3–5 d]

43 Cell resuscitation and passage. Cell preparation was conducted as described in Steps 5–7.

#### Cell starvation and collection [TIMING 11 h]

44 EBSS starvation. The cells were cultured in a 6-cm dish with 4 mL DMEM medium at 37 °C with 5% CO_2_. When the cell density reached around 90%, the medium was discarded, the cells were washed twice with 1 mL PBS, and then starved with 4 mL EBSS medium for 10 h.

#### Extraction of total RNA using Novozan's R401 reagent [TIMING 2–4 h]

45 Washing. Discard the culture medium and wash the cells once with 1 mL 1× PBS.

46 Addition of RNA isolater. Add 1 mL RNA isolater to the cells in a 6-cm dish, ensuring it fully covers the cell surface. Dislodge the cells from the surface using a pipette.

47 Cell Lysis. Transfer the cells to a new 1.5 mL microtube after counting, with a total quantity of 1.5 × 10^6^. Pipette the mixture repeatedly until complete cell lysis is achieved. Incubate the tube on ice for 5 min.

48 Chloroform Addition. Add 1/5 volume of chloroform to the lysate, approximately 200 μL. Vigorously shake the solution for 15 s to ensure thorough mixing and the formation of a milky solution. Incubate the mixture at 4 °C for 5 min.

49 Centrifugation. Centrifuge at 12,000 *g* for 15 min at 4 °C. After centrifugation, the solution will separate into three layers: a clear aqueous layer on top, a white interphase, and a red organic phase at the bottom.

50 RNA Precipitation. Carefully transfer about 400–500 µL of the upper aqueous phase to a new centrifuge tube, avoiding the interphase to prevent genomic DNA contamination. Add an equal volume of pre-chilled isopropanol, around 400–500 μL. Invert the tube several times to mix and incubate at 4 °C for 10 min.

51 RNA Washing. Centrifuge at 12,000 *g* for 10 min at 4 °C. A white RNA pellet should be visible. Carefully discard the supernatant. Add 1 mL of 75% ethanol (prepared with RNase-free water) to the pellet. Tap the tube to disperse the pellet and invert several times. Incubate at RT for 3–5 min.

52 Centrifugation. Centrifuge at 12,000 *g* for 5 min at 4 °C and discard the supernatant as thoroughly as possible. A brief additional spin can help gather residual liquid at the bottom for removal.

53 RNA rehydration. Air-dry the pellet for 5 min at RT in a clean environment. Be careful not to over-dry, as this can make the RNA difficult to dissolve. Rehydrate the RNA pellet in an appropriate amount of RNase-free water, approximately 50–80 μL. If necessary, pipette gently to aid dissolution.

54 Storage. Once fully dissolved, take a small amount for testing, and store the remaining RNA at −80 °C.

55 RNA gel. The experimental procedure is the same as Step 42.

### Detecting yeast ribophagy with TEM

#### Cell culture [TIMING 4 d]

56 Activation and cultivation. Yeast strains: BY4741-Rpl10-APEX2::*HisMX6-pep4*Δ::*KanMX6*, BY4741-Rpl10-Apex2::*HisMX6-atg1*Δ::*hphNT1-pep4*Δ::-*KanMX6*. The experimental procedure is the same as Steps 1 and 2.

#### Yeast starvation and collection [TIMING 7 h]

57 Nitrogen starvation and sample collection. The experimental procedure is the same as Step 3.

**[CRITICAL STEP]** To detect ribophagy levels in yeast cells using the TEM method, it is necessary to knock out the gene PEP4, which encodes acidic hydrolases, thereby inhibiting the degradation of ribosomes within vacuoles.

#### Preparation and analysis of yeast TEM samples [TIMING 2 d]

58 Washing. Wash the collected 20 OD_600_ yeast cells once with 500 μL PBS.

59 Cell fixation. Prepare 1 mL suspension of fixative solution (2% paraformaldehyde and 2.5% glutaraldehyde in PBS). Resuspend the yeast cells by pipetting and shaking them at 200 r/min at 30 °C for 30 min, followed by overnight incubation at 4 °C.

60 Rinsing. The following day, centrifuge at 2000 *g*. Wash the cells three times with 1mL PBS to remove the fixative.

61 DAB staining. Incubate the fixed cells in 1 mL 1× DAB staining buffer at RT, shielded from light, rotating at 100 r/min for 15 min. Stop the staining process by washing twice with PBS. Monitor cell aliquots under a light microscope to track staining progress.

62 Potassium permanganate treatment and thorough washing. Centrifuge to collect the cells and incubate them with 1 mL 2% potassium permanganate at RT for 2 h. Wash the cells five times with 1 mL distilled water to eliminate potassium permanganate.

**[CRITICAL STEP]** The cells should remain black after permanganate treatment and washing.

63 Dehydration. Gradually increasing ethanol concentrations (30%, 50%, 70%, 80%, 90%, 95% each for one round, and 100% twice, using 1 mL for each dehydration step), incubating for 15 min each at RT. Finally, substitute with 1 mL 100% acetone twice for 15 min each.

64 Embedding. Incubate continuously at RT with 500 μL Epon 812-acetone mixed buffer (Epon 812: acetone = 1:2) for 2 h, followed by 500 μL Epon 812-acetone mixed buffer (Epon 812: acetone = 1:1) for 2 h, and then with 500 μL Epon 812-acetone mixed (Epon 812: acetone = 2:1) buffer for 2 h. Finally, replace with fresh 800 μL 100% Epon 812 and allow overnight drying at RT. The next day, replace with fresh 800 μL 100% Epon 812. Place the sample in a 37 °C incubator for 12 h, then transfer to a 65 °C incubator for over 48 h.

65 Sectioning and staining. Use an ultramicrotome to cut thin sections (approximately 60–90 nm). Stain sections with heavy metals such as uranyl acetate and lead citrate to enhance contrast.

66 Examination. Examine the samples under a TEM for result analysis.

### Detecting mammalian ribophagy with TEM

#### Cell culture [TIMING 3–5 d]

67 Cell resuscitation and passage. Cell preparation was conducted as described in Steps 5–7.

#### Cell starvation and collection [TIMING 11 h]

68 EBSS starvation and collection. The experimental procedure is the same as Step 44. Additionally, supplement the EBSS medium with 500 nmol/L Concanamycin A.

**[CRITICAL STEP]** When subjecting mammalian cells to EBSS starvation, adding Concanamycin A inhibits lysosomal acidification, thus preventing the degradation of ribosomes within autolysosomes.

#### Preparation and analysis of mammalian TEM samples [TIMING 2 d]

69 Cell fixation. Cells collected from EBSS medium after 10 h of starvation in a 6-cm dish were washed twice with 1 mL 1× PBS in a 1.5-mL EP tube, and then fixed the cells overnight with 2.5% paraformaldehyde after counting, with a total quantity of 1.5 × 10^6^.

**[CRITICAL STEP]** Take care not to disperse or disrupt the cells during the process. Samples can be stored in a 4 °C refrigerator for several days to weeks. Regularly monitor the fixed liquid and replace it with fresh fixed liquid every 2–4 weeks.

70 Rinsing. Rinse the fixed cells three times with 1 mL 0.1mol/L PBS for 10 min each.

71 Austenic acid and uranium acetate treatment. Add approximately 50–100 μL of 1% austenic acid to the sample for 1 h. Rinse the sample with water 2–3 times, allowing each rinse to last 10 min. Finally, about 100 μL of 2% uranium acetate was added to the sample for 0.5 h.

72 Dehydration. Dehydrate the sample successively with 1mL of 50%, 70%, and 90% ethanol for 15 min each. Subsequently, treat it with 1 mL 100% ethanol for 20 min. Finally, replace with 1 mL 100% acetone twice for 20 min each time.

73 Embedding. Infiltrate and embed the cells in a resin that solidifies for thin sectioning. Begin by reacting the sample with 500 μL mixed buffer A (Epon 812: acetone = 1:1) at RT for 2 h. Follow by reacting the sample with 300 μL mixed buffer B (Epon 812: acetone = 2:1) at RT for 2 h. Conclude by replacing it with 500 μL pure Epon 812 for an overnight reaction. Place the sample in a 37 °C incubator for 12 h, then transfer to a 65 °C incubator for over 48 h.

74 Sectioning and staining. Use an ultramicrotome to slice thin sections (around 60–90 nm). Enhance contrast by staining sections with heavy metals such as uranyl acetate and lead citrate.

75 Examination. Examine the prepared sections under a TEM.

**[?TROUBLESHOOTING]**

Troubleshooting advices can be found in Table 1.

**Table 1:** Troubleshooting table

Step	Problem	Possible reason	Solutions
1	The inoculated yeast is contaminated	Contaminated medium was used	When inoculating yeast, prepare a tube containing only the medium to serve as a negative control
14	(1) The fluorescent signal is weak (2) The fluorescence intensity is too strong (3) No cells found	(1) The concentration of primary or secondary antibody is too low or the incubation time of primary or secondary antibody is too short (2) The concentration of primary or secondary antibody is too high (3) Cells are not fixed or cells are washed off during the process of making fluorescent slides	(1) Increase the concentration of primary or secondary antibodies and extended incubation time of primary or secondary antibodies (2) Reduce exposure time and fluorescence intensity during imaging (3) Cells were fixed with 4% PFA at RT for 15 min or overnight at 4 °C and handle the cell fluorescent slides with care to avoid direct contact between the slides and the pipette tips.
22	Western blot bands appear distorted or contain small bubbles	The membrane and gel are not adhering well	Using a roller to apply appropriate pressure for proper adhesion between the membrane and the gel
28	Western blot bands are weak	The expression level of the target protein or the concentration of primary antibody is too low	Increase the amount of protein loaded or decrease the dilution ratio of the primary antibody
66	Difficulty to observe the image of ribosome accumulation within vacuoles	The activity of acidic hydrolase within the vacuoles was not inhibited	Knock out the gene *PEP4* encoding acidic hydrolases in yeast cells
75	Difficulty to observe the image of ribosome accumulation within lysosomes	Concanamycin A was not added to the EBSS medium	Add 500 nmol/L Concanamycin A to the EBSS medium

## ANTICIPATED RESULTS

### Fluorescence microscope observation of yeast and mammalian ribophagy

**Figure 1:**
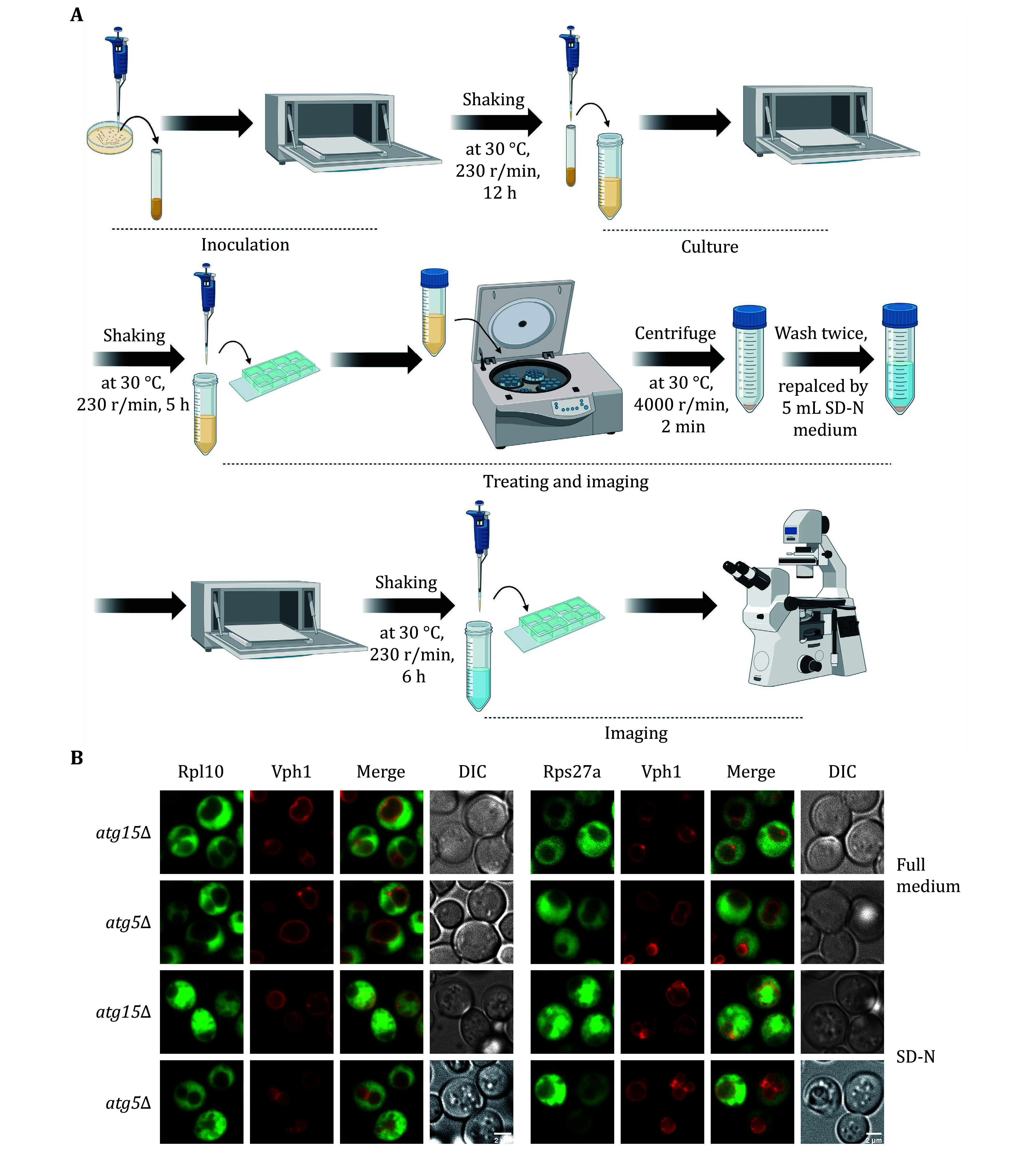
Detecting yeast ribophagy using a fluorescence microscope. **A** Operating procedures for yeast ribophagy using a fluorescence microscope. **B** *atg15*Δ or *atg5*Δ yeast strains co-expressing Vph1-mCherry along with either Rpl10-GFP or Rps27a-GFP were subjected to SD-N for 0 or 6 h. Images of cells were obtained using fluorescence microscopy. Scale bar, 2 μm

**Figure 2:**
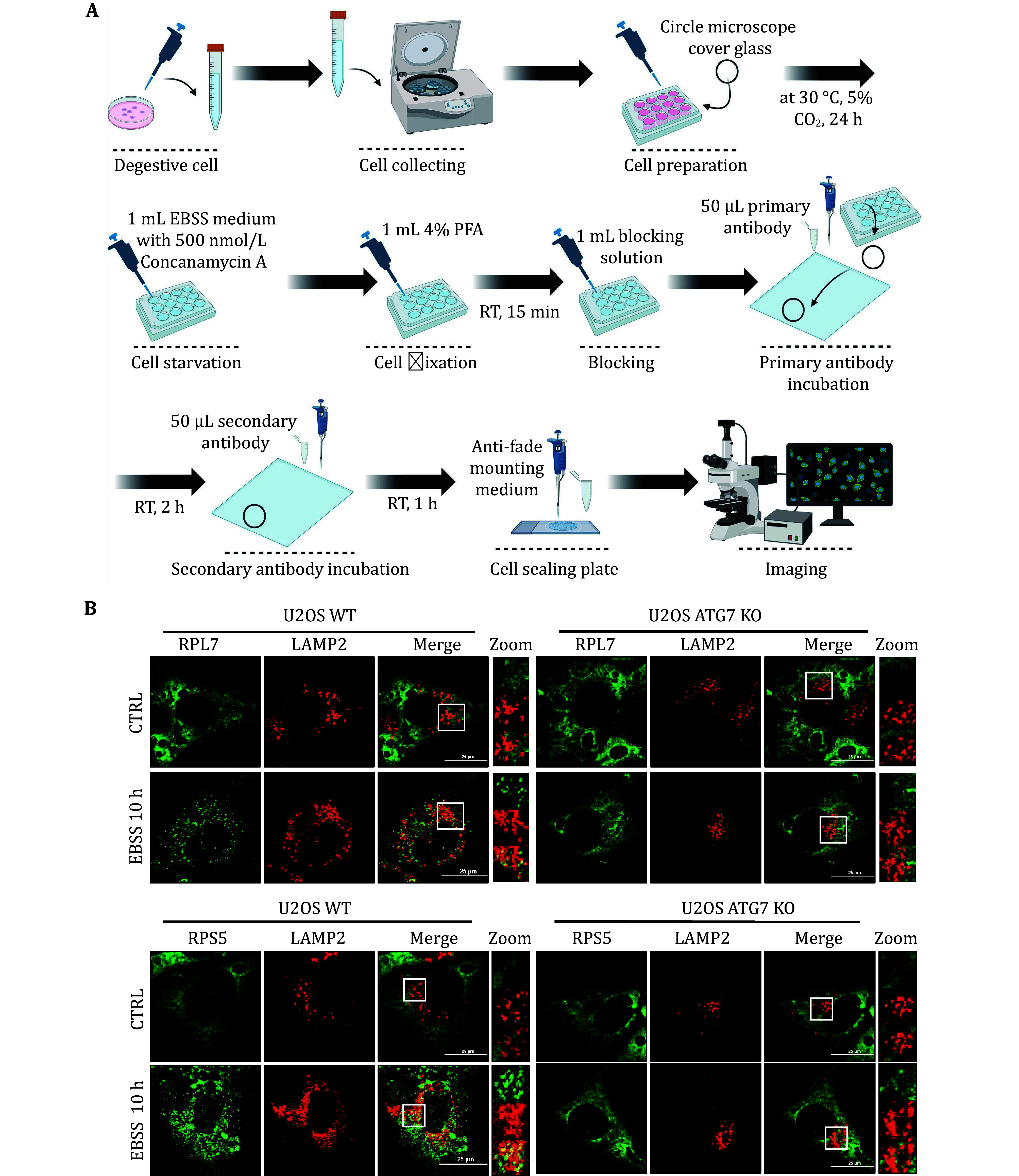
Detecting mammalian ribophagy using a confocal laser scanning microscope. **A** Operating procedures for mammalian ribophagy using a confocal laser scanning microscope. **B** Immunofluorescence staining of lysosomal marker LAMP2 (red) combined with either the ribosome's large subunit RPL7 (green) or the small subunit RPS5 (green), both with EBSS treatment for 10 h, in U2OS WT or ATG7 KO cells. Images of cells were obtained using a confocal laser scanning microscope. Scale bar, 25 μm

We initially assessed ribophagy in both yeast and mammalian cells. Figures 1A and 2A illustrate the process involved in detecting ribophagy in yeast and mammalian cells. Fluorescence microscopy enables the observation of Rpl10-GFP or Rps27a-GFP localization in yeast cells under nutrient-rich or nitrogen starvation conditions (Fig. 1B). *ATG15* is a phospholipase B within the vacuole; its knockout prevents the degradation of autophagic bodies, causing autophagosome accumulation in yeast vacuoles. This accumulation facilitated easier ribophagy observation under a fluorescence microscope. *ATG5* is an essential gene for autophagy. We observed that Rpl10-GFP or Rps27a-GFP did not enter the vacuole under nutrient-rich conditions, indicating that yeast cells do not undergo ribophagy in such conditions. However, in *atg15∆* cells, both Rpl10-GFP and Rps27a-GFP entered the vacuole, while Rpl10-GFP and Rps27a-GFP did not enter the vacuole in *atg5∆* cells under nitrogen starvation conditions. These results indicate that nitrogen starvation can induce ATG5-dependent ribophagy. Subsequently, the laser confocal microscope was employed to observe the localization of Rpl7 or Rps5 in mammalian cells under nutrient-rich or EBSS starvation conditions. To facilitate the observation of ribosomal proteins entering lysosomes during starvation, we introduced Concanamycin A to the EBSS medium to inhibit the lysosomal acidification. Our analysis via laser confocal microscopy revealed that both Rpl7 and Rps5 entered the lysosomes under EBSS starvation conditions. Conversely, *ATG7* KO cells inhibited the lysosomal entry of Rpl7 and Rps5 (Fig. 2B). Notably, the research group led by Dr. J. Wade Harper has developed an RPS3-Keima system for detecting ribophagic flux in mammals (An *et al.* 2018).

### Western blot analysis of yeast and mammalian ribophagy

**Figure 3:**
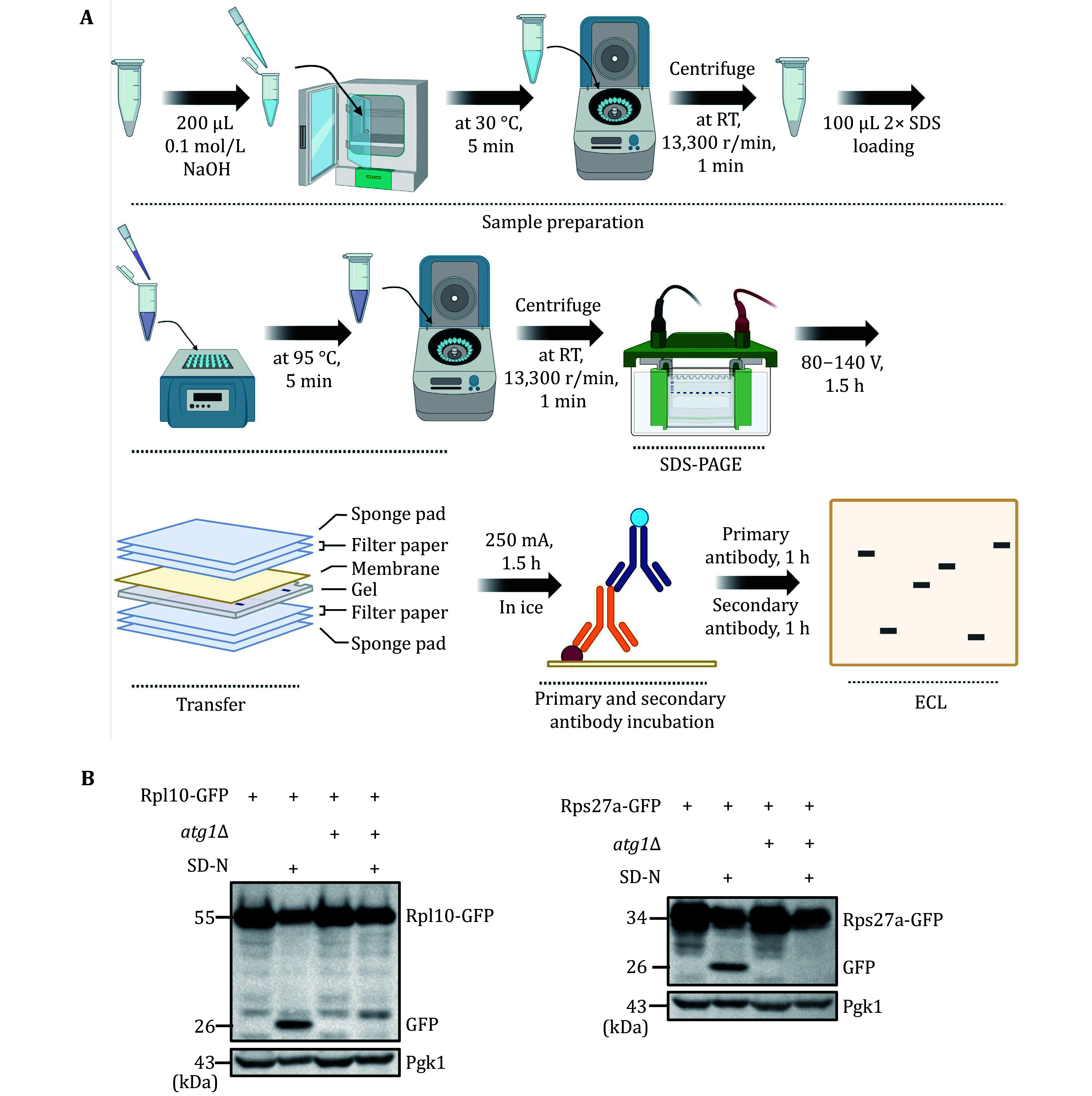
Validating yeast ribophagy by Western blot analysis. **A** The flowchart for validating yeast ribophagy through Western blot analysis. **B** Wild-type (BY4741) or *atg1*∆ yeast cells expressing Rps27a or Rpl10 tagged with GFP were cultured until reaching the logarithmic phase. Subsequently, they were subjected to nitrogen starvation for 0 or 6 h. The GFP cleavage of the specified fusion proteins was detected using an anti-GFP antibody. Pgk1 served as the loading control

**Figure 4:**
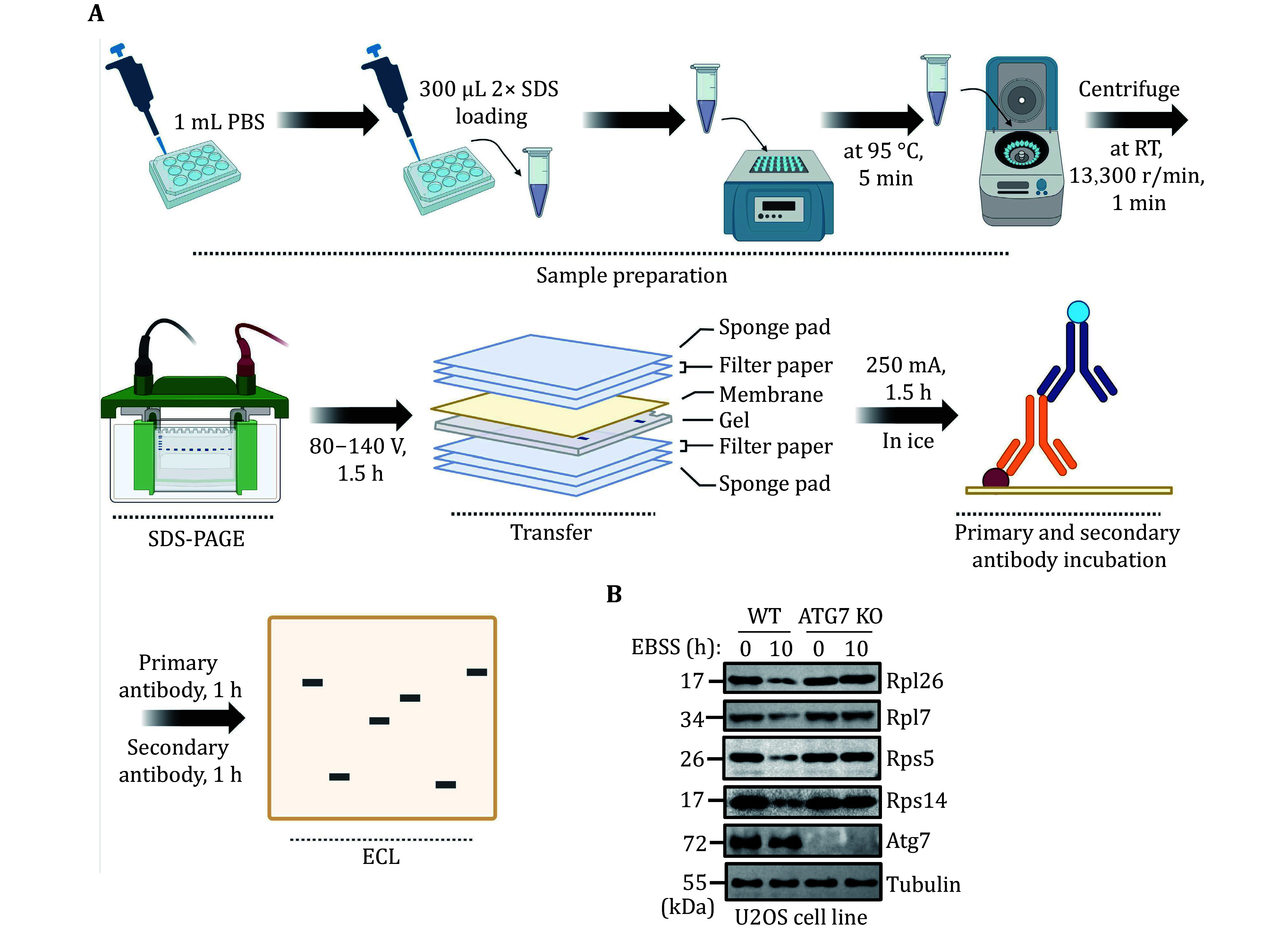
Validating mammalian ribophagy by Western blot analysis. **A** The flowchart for validating mammalian ribophagy through Western blot analysis. **B** U2OS WT or ATG7 KO cells were starved with EBSS for 0 or 10 h. The degradation of the specified ribosomal proteins was detected using their corresponding antibodies. Tubulin served as the loading control

To accurately quantify ribophagy, it is crucial to assess the degradation level of ribosomal proteins using Western blot analysis. Figures 3A and 4A illustrate the process of detecting ribophagy through Western blot analysis in yeast and mammalian cells. In yeast cells, we tagged the target ribosomal proteins, Rpl10 and Rps27a, with GFP tags and analyzed the levels of GFP cleavage in Rpl10-GFP and Rps27a-GFP using Western blot assays. A higher generation of free GFP indicates a more pronounced occurrence of ribophagy. The results indicate that under nitrogen starvation conditions, both Rpl10-GFP and Rps27a-GFP expressed in wild-type yeast cells exhibit free GFP production, signifying the occurrence of ribophagy. However, Rpl10-GFP and Rps27a-GFP no longer produce free GFP in *atg1*∆ cells, indicating the necessity of Atg1 for ribophagy (Fig. 3B). In mammalian cells, due to the availability of commercial ribosomal protein antibodies, we assessed the degradation of corresponding ribosomal proteins under EBSS starvation conditions. Western blot analysis revealed a significant reduction in these ribosomal proteins under EBSS starvation conditions. Conversely, in ATG7 KO cells, there was no notable decrease in ribosomal proteins under EBSS starvation conditions (Fig. 4B), suggesting that the degradation of ribosomal proteins during EBSS starvation relies on autophagy.

### Assessing the degradation of yeast and mammalian rRNA

**Figure 5:**
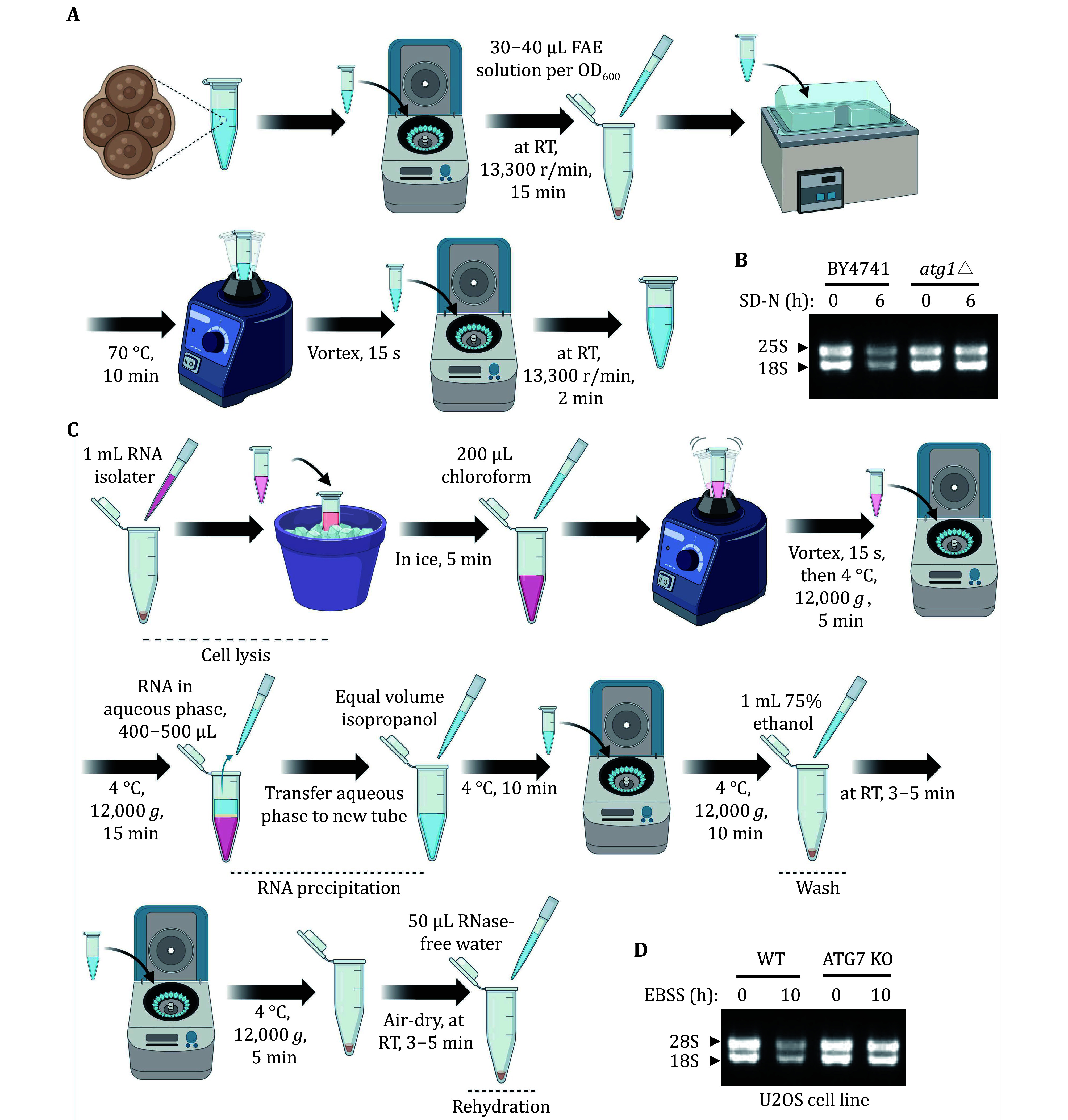
Assessing yeast and mammalian ribophagy through rRNA analysis. **A** The flowchart depicting the process of yeast RNA extraction. **B** Wild-type (BY4741) or *atg1*∆ cells were cultured until reaching the logarithmic phase. Subsequently, they were subjected to nitrogen starvation for 0 or 6 h. After collecting five OD_600_ yeast samples, RNA was extracted and a formaldehyde agarose gel was run. The arrow indicates the corresponding 25S and 18S rRNA, respectively. **C** The flowchart depicting the process of mammalian RNA extraction. **D** When the U2OS WT or ATG7 KO cells on the 6-cm dish reached a density of 90%, they were starved with EBSS for 0 or 10 h. Total RNA was extracted and a formaldehyde agarose gel was run. RNA from equal numbers of cells was loaded in each lane. The arrowhead indicates that the corresponding 28S and 18S rRNA, respectively

Considering that ribosomes consist of numerous ribosome-related proteins and rRNA, the degradation level of rRNA is a crucial measure for assessing the occurrence of ribophagy. Figures 5A and 5C illustrate the process of detecting rRNA level through total RNA extraction in both yeast and mammalian cells. In yeast cells, we isolated total RNA from both wild-type and *ATG1* KO cells. Our analysis using RNA agarose gel revealed that, under nitrogen starvation conditions, the levels of rRNA notably decreased in wild-type yeast cells compared to those in nutrient-rich conditions. Conversely, the degradation of rRNA was impeded in *atg1*∆ cells (Fig. 5B). This observation implies that autophagy regulates yeast rRNA degradation. Additionally, other yeast total RNA extraction methods, such as the hot acidic phenol method, are also applicable for yeast total RNA extraction. In mammalian cells, total RNA extraction from U2OS WT and ATG7 KO cells. Subsequent RNA agarose gel analysis demonstrated a significant reduction in rRNA levels under EBSS starvation conditions in U2OS WT cells compared to nutrient-rich conditions. In contrast, ATG7 KO cells blocked the degradation of rRNA (Fig. 5D), indicating that autophagy-mediated the degradation of rRNA is conserved from yeast to humans.

### TEM analysis of yeast and mammalian ribophagy

**Figure 6:**
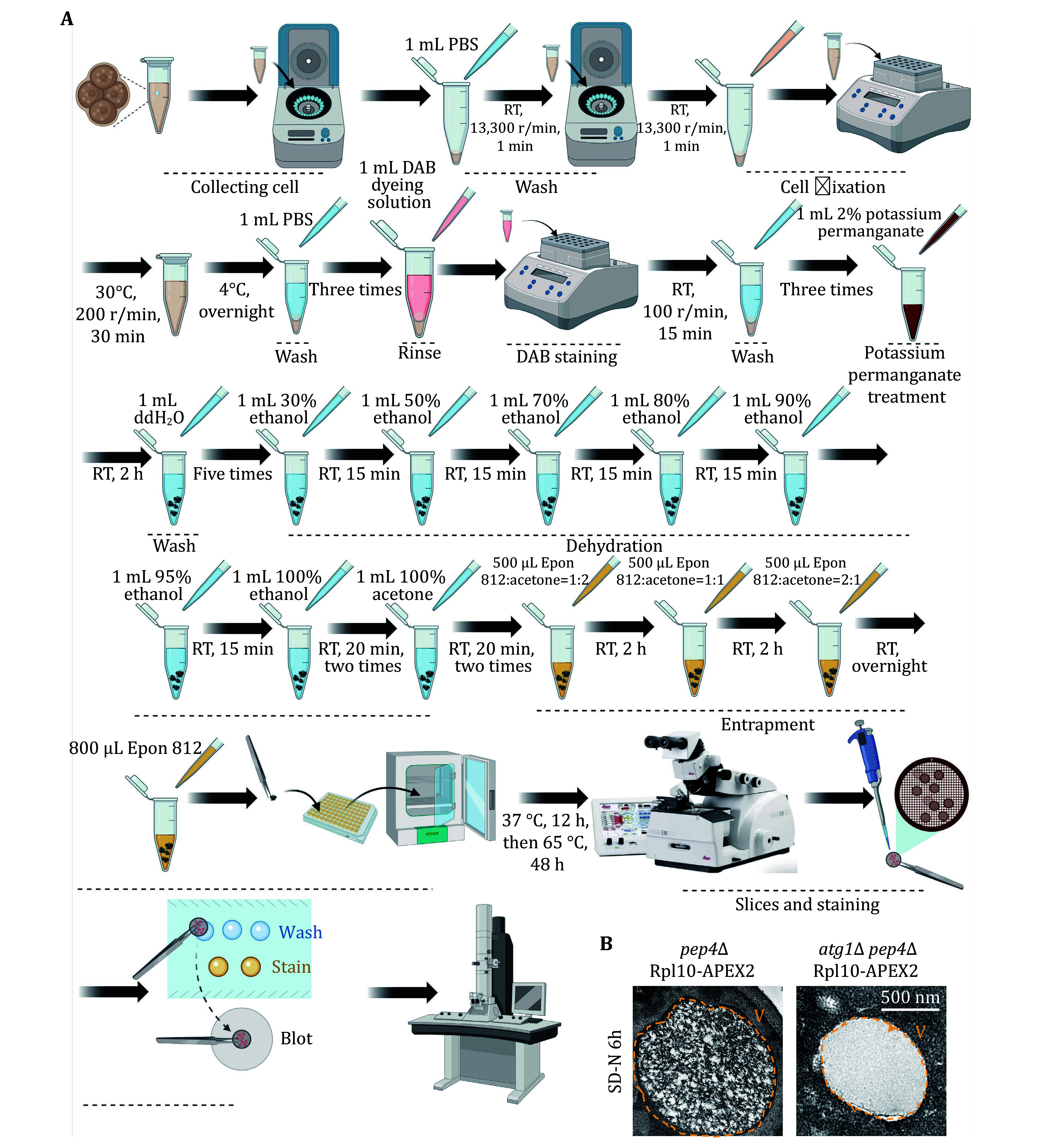
Detecting yeast ribophagy using TEM. **A** The flowchart illustrates the detection of yeast ribophagy using TEM. **B** *pep4∆* or *atg1∆-pep4∆* yeast cells expressing Rpl10-APEX2 were cultured until they reached the logarithmic phase. Subsequently, they were subjected to nitrogen starvation for 6 h. Following the preparation of the samples, the occurrence of ribophagy was observed through TEM. V: Vacuole. Scale bar: 500 nm

**Figure 7:**
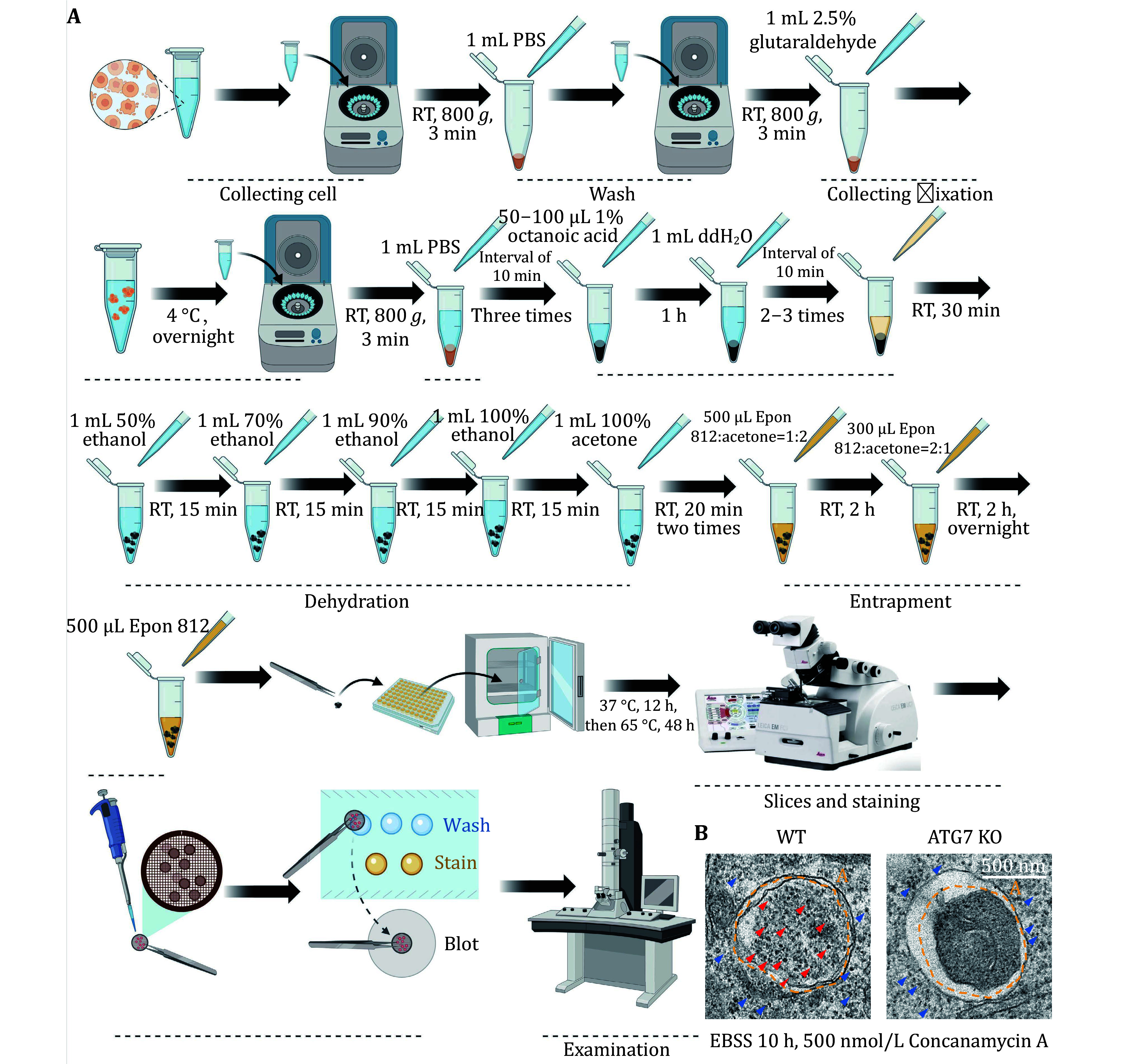
Detecting mammalian ribophagy using TEM. **A** The flowchart illustrating the detection of mammalian ribophagy using TEM. **B** When the U2OS WT or ATG7 KO cells on the 6-cm dish reached a density of 90%, they were treated with EBSS and concanamycin A for 10 h, and the samples were collected. Following sample preparation, the occurrence of ribophagy was observed through TEM. The black dot in the picture represents a ribosome. Red arrowheads indicate ribosomes within an autolysosome. Blue arrowheads indicate ribosomes in the cytoplasm. A: Autolysosome. Scale bar: 500 nm

TEM is the gold standard method for detecting autophagy. Hence, we utilized TEM techniques to assess the occurrence level of ribophagy. Figures 6A and 7A illustrate the process of detecting ribophagy through TEM assays in both yeast and mammalian cells. In yeast cells, we tagged the ribosomal large subunit protein Rpl10 with an APEX2 tag at the C-terminus, labeling yeast ribosomes with Rpl10-APEX2. TEM analysis revealed significant ribosome accumulation in the vacuoles of *pep4*∆ yeast cells under nitrogen starvation conditions, while no ribosomes were present in the vacuoles of *atg1*∆ yeast cells (Fig. 6B), indicating that nitrogen starvation induces ribophagy, which is Atg1-dependent. For mammalian cells, we supplemented the EBSS medium with Concanamycin A. TEM analysis showed abundant ribosomal particles within autolysosomes under EBSS starvation conditions, whereas in ATG7 KO cells, ribosomal particles did not enter the autolysosomes (Fig. 7B), indicating that EBSS starvation induces ribophagy, which is Atg7-dependent.

## MATERIALS

### Biological materials

All yeast strains used in this work were originated from the wild type (BY4741) or related deletion strains. The wild type and related deletion strains were purchased from Invitrogen (95,401.H2).

U2OS WT and ATG7 KO cells were donated by Professor Cui Yixian from Wuhan University.

### Reagents

• Yeast extract (Sangon Biotech, cat. no. A515245)

• Tryptone (Sangon Biotech, cat. no. A505250)

• Agar (BioFroxx, cat. no. 8211KG001)

• Yeast nitrogen base (without amino acids and ammonium sulfate) (BD, cat. no. 233510)

• (NH_4_)_2_SO_4_ (Sangon Biotech, cat. no. A100191)

• Glucose (Sangon Biotech, cat. no. A100188)

• Adenine (BBI Life Sciences, cat. no. A600013)

• L-Arginine (BBI Life Sciences, cat. no. A610206)

• L-Aspartic acid (BBI Life Sciences, cat. no. A600091)

• L-Isoleucine (BBI Life Sciences, cat. no. A600914)

• L-Leucine (BBI Life Sciences, cat. no. A600922)

• L-Lysine (BBI Life Sciences, cat. no. A602759)

• L-Methionine (BBI Life Sciences, cat. no. A610346)

• L-Phenylalanine (BBI Life Sciences, cat. no. A610422)

• L-Threonine (BBI Life Sciences, cat. no. A610919)

• L-Tryptophan (BBI Life Sciences, cat. no. A601911)

• L-Tyrosine (BBI Life Sciences, cat. no. A601932)

• L-Valine (BBI Life Sciences, cat. no. A600172)

• Uracil (BBI Life Sciences, cat. no. A610564)

• Hygromycin B (YEASEN, cat. no. 60225ES10)

• G418 (YEASEN, cat. no. 60220ES08)

• NaOH (BBI Life Sciences, cat. no. A100173)

• SDS (Solarbio Life Sciences, cat. no. S8010)

• Tris (Sangon Biotech, cat. no. A610195)

• HCl (Hushi, cat. no. 10011018)

• Glycerol (Sangon Biotech, cat. no. A501745)

• Bromophenol blue (Sangon Biotech, cat. no. A500922)

• β-Mercaptoethanol (Macklin, cat. no. M6230)

• Glycine (Sangon Biotech, cat. no. A502065)

• Methanol (Hushi, cat. no. 100141190)

• NaCl (Sangon Biotech, cat. no. A610476)

• KCl (Sangon Biotech, cat. no. A501159)

• KH_2_P0_4_ (BBI Life Sciences, cat. no. A600445)

• Na_2_HP0_4_ (BBI Life Sciences, cat. no. A610404)

• Tween-20 (BBI Life Sciences, cat. no. A600560)

• Skimmed milk. (Beyotime, cat. no. P0216)

• Primary antibody dilution buffer (Beyotime, cat. no. P0023A)

• Fetal Bovine Serum, Qualified (VISTECH, cot. no. SE200-ES)

• EBSS medium (Solarbio, cat. no. H2025)

• DMEM (Gibco, cot. no. 12100061)

• Trypsin (YEASEN, cat. no. 40101ES25)

• Penicillin-Streptomycin for Cell Culture (Beyotime, cat. no. ST488S)

• EDTA (Sangon Biotech, cat. no. A600075-0500)

• Antibody (anti-GFP antibody (Proteintech, cat. no. 50430-2-AP); anti-Pgk1 antibody (homemade); anti-RPL7, Proteintech, cat no. 14583-1-AP; anti-RPS5, Proteintech, cat no. 16964-1-AP; anti-RPL26, Proteintech, cat no. 17619-1-AP; anti-RPS14, Proteintech, cat no. 16683-1-AP; anti-Lamp2, Proteintech, cat. no. 27823-1-AP; anti-Atg7, Proteintech, cat. no. 10082-2-AP; anti-Tubulin, Proteintech, cat. no. 11224-1-AP; Goat anti-Mouse IgG H&L Alexa Fluor^®^ 488, Abcam, cat. no. ab150113; Goat anti-Rabbit IgG H&L Alexa Fluor^®^ 594, Abcam, cat. no. ab150080)

• Anti-fade mounting medium (Sangon Biotech, cat. no. E675011)

• Glutaraldehyde (Hushi, cat. no. 30092436)

• Austenic acid (Sangon Biotech, cat. no. A600055)

• Ethanol (Hushi, cat. no. 10009128)

• Acetone (Hushi, cat. no. 10000418)

• Epon 812 (Sigma, cat. no. 45359)

• Lead citrate (Sigma, cat. no. 15326)

• Uranium acetate (FangXinShengWu, cat. no. F00022)

• Uranyl acetate (YaJi Biological, cat. no. YS25690U)

• Formamide (Sigma, cat. no. F9037)

• RNA Isolater (Vazyme, cat. no. R401-01)

• Chloroform (Hushi, cat. no. 10006818)

• Isopropanol (Hushi, cat. no. 80109218)

### Reagent setup

• Yeast medium amino acids and vitamins mixture without Histidine. Mix 10 mg Adenine, 50 mg L-Arginine, 80 mg L-Aspartic acid, 50 mg L-Isoleucine, 100 mg L-Leucine, 50 mg L-Lysine, 20 mg L-Methionine, 50 mg L-Phenylalanine, 100 mg L-Threonine, 50 mg L-Tryptophan, 50 mg L-Tyrosine, 140 mg L-Valine and 20 mg Uracil for 1 L SD-His medium.

• Yeast medium amino acids and vitamins mixture. Mix 10 mg Adenine, 50 mg L-Arginine, 80 mg L-Aspartic acid, 20 mg L-Histidine, 50 mg L-Isoleucine, 100 mg L-Leucine, 50 mg L-Lysine, 20 mg L-Methionine, 50 mg L-Phenylalanine, 100 mg L-Threonine, 50 mg L-Tryptophan, 50 mg L-Tyrosine, 140 mg L-Valine and 20 mg Uracil for 1 L SD medium.

• SD-His medium. Dissolved 1.7 g Yeast nitrogen base without amino acids and ammonium sulfate, 20 g glucose, 5 g ammonium sulfate and 0.69 g Yeast medium amino acids and vitamins mixture without Histidine in 900 mL deionized water and stir until it is dissolved. Bring the volume to 1 L and moist-heat-sterilize the solution. This solution can be stored at RT.

• SD medium. Dissolved 1.7 g Yeast nitrogen base without amino acids and ammonium sulfate, 20 g glucose, 5 g ammonium sulfate and 0.79 g Yeast medium amino acids and vitamins mixture in 900 mL deionized water and stir until it is dissolved. Bring the volume to 1 L and moist-heat-sterilize the solution. This solution can be stored at RT.

• SD-N medium. Dissolved 1.7 g Yeast nitrogen base (without amino acids and ammonium sulfate) and 20 g glucose in 900 mL deionized water and stir until it is dissolved. Bring the volume to 1 L and moist-heat-sterilize the solution. This solution can be stored at RT.

• 200 mg/mL Hygromycin B stock. Dissolved 20 g Hygromycin B in 90 mL deionized water and stir until it is dissolved. Bring the volume to 100 mL and filter-sterilize the solution. This solution can be dispensed into 1.5 mL test tubes and stored at −20 °C.

• 250 mg/mL G418 stock. Dissolved 25 g G418 in 90 mL deionized water and stir until it is dissolved. Bring the volume to 100 mL and filter-sterilize the solution. This solution can be dispensed into 1.5 mL test tubes and stored at −20 °C.

• 1 mol/L NaOH stock. Dissolved 40 g NaOH in 900 mL deionized water and stir until it is dissolved. Bring the volume to 1 L. This solution can be stored at RT.

• 0.1 mol/L NaOH. Dilute 10 mL 1 mol/L NaOH stock with 90 mL deionized water.

• 1 mol/L Tris-HCl (pH 6.8) stock. Dissolve 121.14 g Tris in 900 mL deionized water and stir until it is dissolved. Adjust the pH to 6.8 with HCl. Bring the volume to 1 L and moist-heat-sterilize the solution. This solution can be stored at RT.

• 2× SDS sample buffer. Dissolved 40 g SDS and 0.2 g Bromophenol blue in 500 mL deionized water and stir until it is dissolved. Add 120 mL 1 mol/L Tris-HCl (pH 6.8) stock, 100 mL β-mercaptoethanol and 20 mL glycerol into the solution. Bring the volume to 1 L and filter-sterilize the solution. This solution can be dispensed into 1.5 mL test tubes and stored at −20 °C.

• 10× SDS electrophoresis buffer stock. Dissolved 30 g Tris, 144 g glycine and 10 g SDS in 900 mL deionized water and stir until it is dissolved. Bring the volume to 1 L. This solution can be stored at RT.

• SDS electrophoresis buffer. Dilute 100 mL 10× SDS electrophoresis buffer stock with 900 mL deionized water.

• 10× Trans buffer stock. Dissolved 15.15 g Tris and 72 g glycine 10 g SDS in 900 mL deionized water and stir until it is dissolved. Bring the volume to 1 L. This solution can be stored at RT.

• Trans buffer. Add 100 mL 10× Trans buffer stock and 200 mL Methanol to 700 mL deionized water.

• 10× PBS stock. Dissolved 80.06 g NaCl, 2.01 g KCl, 2.72 g KH_2_P0_4_ and 14.2 g Na_2_HP0_4_ in 900 mL deionized water and stir until it is dissolved. Bring the volume to 1 L and moist-heat-sterilize the solution. This solution can be stored at RT.

• PBS. Dilute 100 mL 10× PBS with 900 mL deionized water.

• PBST. Add 0.1% Tween-20 to PBS.

• 5% skimmed milk. Add 2.5 g skimmed milk to 50 mL PBST.

• 10 KU/mL Penicillin. 600 KU/bottle of penicillin powder is dissolved in 100 mL of deionized water to a volume of 160 mL, filtered and sterilized, diluted ten times and added to DMEM medium. This solution can be stored at −20 °C.

• 10 mg/mL Streptomycin. 1 g/bottle of streptomycin powder was dissolved in 80 mL of deionized water, the volume was fixed to 100 mL, filtered and sterilized, and diluted ten times to add to DMEM medium. The prepared mother liquor can be stored at −20 °C.

• DMEM medium. One bottle of DMEM powder medium, 37 g NaHCO_3_, 9 L dissolved in water, HCl adjusted pH to 7.2–7.4, fixed volume to 10 L, filtered and sterilized. Save at 4 °C.

• 2.5% Trypsin solution. 2.5 g trypsin dissolved in 80 mL PBS, adjust the pH at 7.4–7.6, and set the volume to 100 mL for 2.5% trypsin concentrate, which can be filtered and sterilized and stored at −20 °C.

• 0.25% Trypsin-EDTA. Take 10 mL of the 2.5% trypsin concentrate, 5 mL of 2% EDTA, PBS to 500 mL, filter and sterilize. Aliquot cryopreservation.

• 2% EDTA. 1 g EDTA, dissolve in 40 mL of water, set the volume to 50 mL, filter and sterilize.

• PBS. 8.0 g NaCl, 0.2 g KCl, 1.44 g Na_2_HPO_4_ and 0.24 g KH_2_PO_4_ were dissolved in 800 mL of distilled water, the solution was adjusted to 7.4 with HCl, and finally distilled water was added to the volume to 1 L. Filter, 121 °C, 15 min autoclaving.

• 4% PFA. 4 g PFA dissolved in 80 mL PBS, set to 100 mL, filtered, and stored at 4 °C.

• Blocking solution. 0.1% Triton X-100 and 5% FBS in PBS.

• Primary antibody. the blocking solution contained 1:300 RPL7/RPS5 antibodies and 1:1000 Lamp2 antibodies.

• Secondary antibody. the blocking solution contained 1:1000 goat anti rabbit Alexa Fluor 594 and 1:1000 goat anti-rat Alexa Fluor 488.

• 2.5% Glutaraldehyde. Add 2.5% Glutaraldehyde to PBS.

• 1% Austenic acid. Add 1% Austenic acid to deionized water and filter-sterilize the solution.

• 1% Uranium acetate. Add 2% Uranium acetate to deionized water and filter-sterilize the solution.

• 0.5 mol/L EDTA. Dissolved 11.8 g EDTA in 90 mL deionized water and adjust the pH to 8.0 with NaOH, stir until it is dissolved. Bring the volume to 100 mL and filter-sterilize the solution.

• FAE solution. Mix 49.5 mL Formamide and 500 μL 0.5 mol/L EDTA.

• 75% Ethanol. Mix 37.5 mL Ethanol and 12.5 mL deionized water.

## EQUIPMENT

• Pipettes: 10 μL, 200 μL, 1 mL and 5 mL.

• 15-mL Centrifuge tube (Saining Life Science, cat. no. 3030100)

• 50-mL Centrifuge tube (Saining Life Science, cat. no. 3031100)

• 1.5-mL microtube (Axygen, cat. no. MCT-150-C)

• Chambered Cover glass (Cellvis, cat. no. C8-1-N)

• Vertical large-capacity constant temperature shaking incubator (Minquan, MQL-621HR)

• Spectrophotometer (Eppendorf, BioSpectrometer D30)

• Benchtop centrifuge (Eppendorf, Centrifuge 5804 R)

• Benchtop centrifuge for 1.5-mL tube (Thermo Fisher Scientific, Sorvall Legend Micro 17)

• Dry thermostat (Haimen Kylin-Bell Lab Instruments, GL-150B)

• Electrophoresis tank (Tanon, VE-180)

• Transfer electrophoresis tank (Tanon, VE-186)

• Shaker (Haimen Kylin-Bell Lab Instruments, TS-1000)

• Inverted fluorescence microscope (Olympus, IX83)

• Biological safety cabinet (Shanghai Lishen Scientific Instrument, HFsafe-1200LC(A1))

• Carbon dioxide incubator (Shanghai Lishen Scientific Instrument, HF90/HF240)

• Microscope (Motic, AE2000)

• High speed centrifuge (Thermo Fisher SCIENTIFIC, 75002430)

• Medical centrifuge (Thermo Fisher SCIENTIFIC, 75007201-C)

• Water bath (JOANLAB, WB100-2)

• 60-mm cell and tissue culture plates (BIOFIL, TCD000060)

• 100-mm cell and tissue culture plates (BIOFIL, TCD010100)

• Cell and tissue culture plates 12 well (BIOFIL, TCP001012)

• Filter (BIOFIL, FMC201500)

• Microscope slides (CITOTEST, 10127105P-G)

• Circle microscope cover glass (NEST, 801010)

• Mini desktop vacuum pump (Kylin-Bell, GL-802B)

• Nikon Confocal Microscope (Nikon, Ti2 Microscope)

• Electrothermal Constant Temperature Incubator (Shanghai Yiheng Scientific Instruments, cat. no. DHP-9052)

• Frozen ultra-thin slicer (Leica, cat. no. EM UC7)

• Transmission electron microscope (Thermo Fisher SCIENTIFIC, Tecnai G2 spirit)

• Water bath (JOANLAB, WB100-2)

• Vortex meter (Haimen Kylin-Bell Lab Instruments, Vortex-5)

• Centrifuge (Eppendorf, 5424R)

• Gel imager system (Clinx Science Instruments, GenoSens 2100)

• Horizontal nucleic acid electrophoresis tank (Tanon, HE-120)

## Conflict of interest

Miao Ye, Yuting Chen, Zhaojie Liu, Yigang Wang and Cong Yi declare that they have no conflict of interest.
